# Thiamine as a metabolic resuscitator in septic shock: a meta-analysis of randomized controlled trials with trial sequential analysis

**DOI:** 10.3389/fmed.2023.1223862

**Published:** 2023-09-13

**Authors:** Frédéric Sangla, Thomas Verissimo, Anna Faivre, Térence Glauser, Saw Kian Cheah, Benjamin Assouline, Sebastian Sgardello, David Legouis

**Affiliations:** ^1^Intensive Care Medicine Unit, Division of Intensive Care, Department of Acute Medicine, University Hospital of Geneva, Geneva, Switzerland; ^2^Laboratory of Nephrology, Department of Medicine and Cell Physiology, University Hospital and University of Geneva, Geneva, Switzerland; ^3^Department of Medicine, University Hospital of Geneva, Geneva, Switzerland; ^4^Department of Anesthesiology, Centre Hospitalier de Bienne, Bienne, Switzerland; ^5^Department of Anesthesiology and Intensive Care, Hospital Canselor Tuanku Muhriz UKM, Kuala Lumpur, Malaysia; ^6^Department of Surgery, Centre Hospitalier du Valais Romand, Sion, Switzerland

**Keywords:** thiamine, sepsis, septic shock, intensive care medicine, thiamine (B1)

## Abstract

**Objective:**

Septic shock is one of the most common reasons for admission to the Intensive Care Unit (ICU) and is associated with high mortality. Fundamentally, its management rests on antibiotics, fluid therapy and vasopressor use while many adjunctive therapies have shown disappointing results. Thiamine has recently gained interest as a metabolic resuscitator, though recent trials have tempered this enthusiasm, more specifically when thiamine is associated with ascorbic acid. However, thiamine use alone has been poorly investigated.

**Design:**

We conducted a systematic review and meta-analysis of randomized controlled trials (RCTs) in septic shock patients to assess the effects of thiamine without ascorbic acid as an adjunctive therapy.

**Setting:**

PubMed, Embase and the Cochrane library databases were searched from inception to April of 2023. Data were extracted independently by two authors. The main outcome was mortality.

**Subjects:**

We included RCTs comparing standard care using thiamine alone, to standard care or placebo, in patients admitted to the ICU with sepsis or septic shock.

**Main results:**

We included 5 RCTs (*n* = 293 patients). In this analysis, use of thiamine alone did not significantly change mortality, RR 0.87 (95%CI 0.65; 1.16, *I*^2^ = 21%) *p* = 0.34.

**Conclusion:**

Current RCTs did not show an improvement in mortality when using thiamine in septic shock patients as an adjunctive therapy. However, these trials are largely underpowered for a definitive conclusion to be drawn. Further studies are therefore needed to assess the effects of thiamine without ascorbic acid as an adjunctive therapy.

## 1. Background

Septic shock is a common cause of Intensive Care Unit (ICU) admission. This syndrome, characterized by a deregulated host response to infection, leads to organ failure and is associated with mortality (1).

Current septic shock management includes the control of the infection through antimicrobial administration, fluid resuscitation and vasopressor use (2, 3). This management has not evolved over the years, with many trials of adjunctive therapies having failed to demonstrate any benefit.

Following the publication of a retrospective before/after study, which reported dramatic improvement in septic patients’ outcomes, enthusiasm for the use of a combination of thiamine and ascorbic acid has recently increased (4). However, these promising results have not been confirmed in subsequent Randomized Controlled Trials (RCTs) (5).

In parallel the use of thiamine alone, and not in association with ascorbic acid, has recently gained interest, in part due to physiological considerations (6). However, RCTs assessing the effects of thiamine used alone in septic ICU patients are scarce. Moreover, the few existing studies have enrolled only a small number of patients, thus limiting their clinical significance.

We conducted a systematic review and meta-analysis of RCTs with trial sequential analysis to investigate the effects of thiamine in sepsis as well as taking into account the risk of type 1 errors due to the small number of trials relating to this topic.

## 2. Methods

### 2.1. Protocol and registration

This review complies with the PRISMA statement (7). Ethical approval was waived by the local Ethics Committee (Commission Cantonale d’Ethique de la Recherche, Req-2021-00256).

### 2.2. Information sources and search

MEDLINE, EMBASE and the Cochrane Library were searched, without language restriction, using the following terms: [(thiamine) OR B1) AND ((sepsis) or (septic shock)]. Further, additional filters were applied to restrict the results to RCTs. Finally, we manually selected the studies focusing on septic shock patients. Searches were re-run before the final analysis (April 10th 2023). References from retrieved articles were reviewed for additional studies.

### 2.3. Eligibility criteria

We included RCTs in adult patients (>18 years of age) admitted to an ICU for septic shock that assessed the effects of thiamine. To be included in the treatment group, the patients must have had received standard of care for septic shock (3) and thiamine. Exclusion criteria were the following: population other than septic shock patients, studies using thiamine in combination with other adjunctive therapies (mainly ascorbic acid) and studies published as abstracts only.

### 2.4. Study selection

Two authors screened for titles and abstracts of potentially relevant records and independently selected the studies based on a full-text review. Disagreements were resolved by consensus.

### 2.5. Data collection process

For each included study, one author extracted the first author’s name, year of publication, setting, patient’s characteristics and comorbidities, study site and prespecified outcomes. Another author independently verified the extracted data.

### 2.6. Data items

The primary endpoint was overall mortality. Secondary endpoints included SOFA reduction within 4 days and need for renal replacement therapy (RRT).

### 2.7. Risk of bias in individual studies

All studies were assessed for the risk of bias using the Cochrane risk of bias tool and GRADEpro GDT (GRADEpro Guideline Development Tool [Software], McMaster University). Using the Cochrane tool, two investigators independently carried out a risk of bias assessment based on the primary outcome (mortality). Disagreements were resolved through discussion. Included trials were rated as low risk of bias when 5 domains were judged as having a low risk of bias. The Robvis tool was used to create the risk of biases plots (8).

### 2.8. Summary measures and synthesis of results

Meta-analyses were performed if data from at least 3 trials or 100 patients could be combined.

We reported the risk ratio (RR) for mortality with a 95% CI. The effect estimates were computed for each individual study and were combined into a pooled weighted estimate using Mantel–Haenszel weights. In case of zero events, a constant continuity correction was applied by adding 0.5 to each cell. Fixed effect model was used to combine homogenous data. Heterogeneity was estimated using *I*^2^ and Tau^2^ statistics (*p* < 0.1). Publication bias was assessed graphically using funnel plots.

We performed TSA with O’Brien-Fleming alpha-spending boundaries to identify the minimum required information size (RIS) to verify our hypothesis. As assumptions, we considered 40% mortality in the control group, an *a priori* relative risk reduction of 14% in the thiamine group, a power of 80% and an alpha-risk of 5%.

All analyses were performed using RevMan 5.4 (Cochrane Collaboration, Oxford, UK) and TSA software (v0.9, beta software, Copenhagen Trial Unit, Centre for Clinical Intervention Research, Copenhagen, Denmark).

## 3. Results

### 3.1. Selection of studies

**Figure 1:**
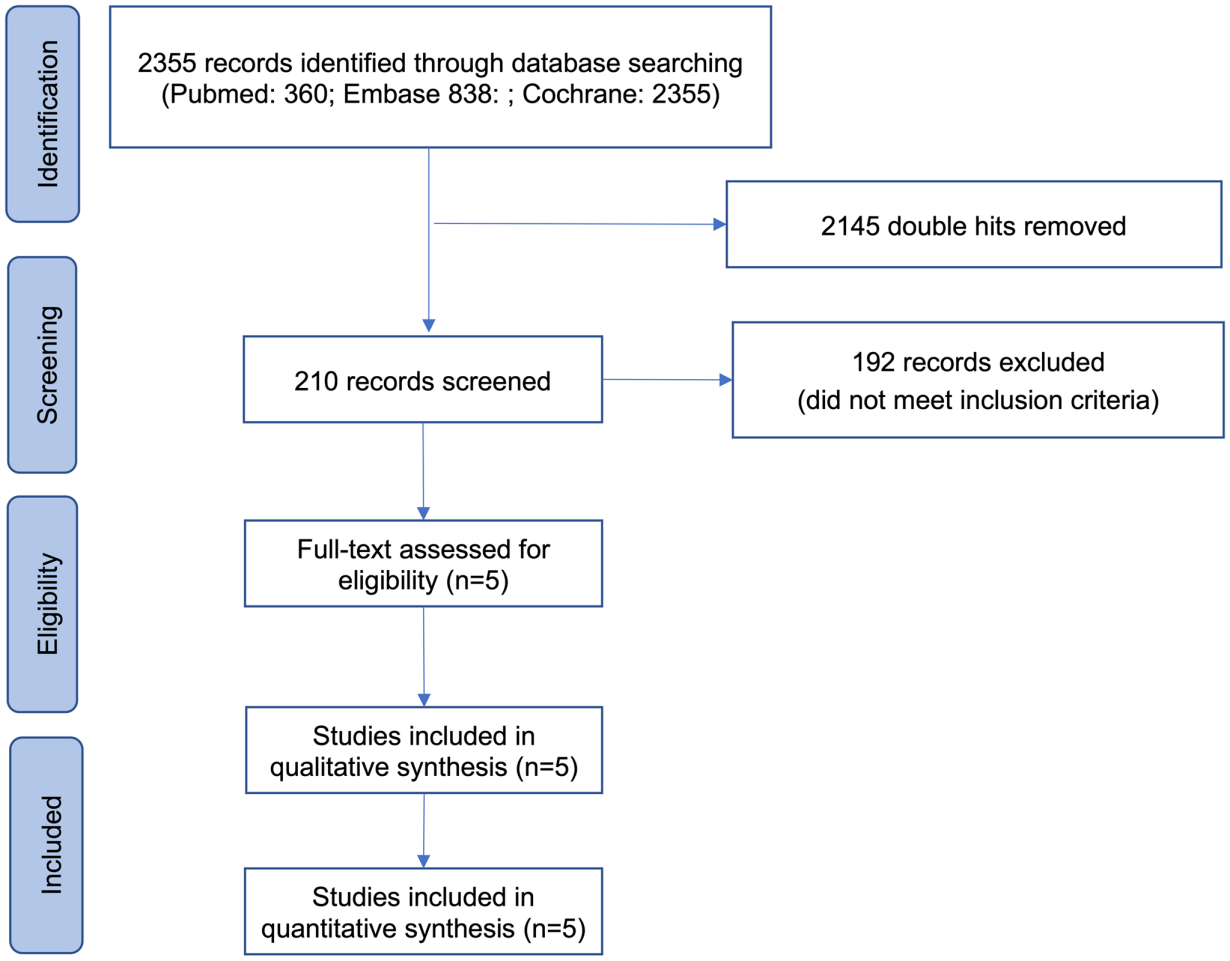
PRISMA flow diagram for the systematic review and meta-analysis.

After removing 2,145 double hits, the search identified 210 unique records of which 192 did not fulfill the inclusion criteria. Hence, 5 studies involving 293 patients were included in the quantitative analysis (Figure 1).

### 3.2. Risk of bias

A summarized risk of bias assessment for studies reporting ICU mortality is presented in Supplementary file 1. Four out of 5 studies were rated as having a low risk of bias when assessing the primary outcome. Some concerns about a risk of bias were found in 1 study.

### 3.3. Study characteristics

The main characteristics of the study population are summarized in Table 1. The RCTs were published between 2016 and 2022 and conducted in 5 different countries. The median number of patients per study was 50 (range 40–88). Drug doses in the intervention group were consistent in all trials with a median of 400 mg per day.

**Table 1:** Characteristics of the included studies.

Study/year	Design	Sample size (thiamine/placebo)	Age, mean (thiamine/placebo)	Male, *n* (%) (thiamine/placebo)	Experimental intervention	Primary outcome	Secondary outcomes
Donnino et al. (9)	Two-center randomized controlled trial	43/45	70/65	60/58	IV thiamine 200 mg, every 12 h, for 7 days or until hospital discharge	Lactate level 24 h after the first study medication dose	Lactate levels at 6 and 12 h Lactate change at 24 h Time to shock reversal APACHE II score at 24 h SOFA score at 24 h ICU and hospital length of stay In-hospital mortality
Harun et al. (10)	Single-center randomized controlled trial	32/33	63/67	62/55	IV thiamine 200 mg, every 8 h, for 3 days	Lactate changes over 24 h	Time for shock reversal Changes of the SOFA score over 72 h ICU length of stay ICU mortality
Petsakul et al. (11)	Single-center randomized controlled trial	25/25	64/66	68/48	IV thiamine 200 mg, every 12 h, for 7 days	Vasopressor-free days over 7 days	Lactate reduction within 24 h after administration of thiamine Vasopressor dependency index reduction within 24 h after administration of thiamine Changes in the vasopressor dependency index from baseline to day 7 Changes in SOFA scores from baseline to day 7 28-day mortality
Ap et al. (12)	Single-center randomized controlled trial	20/20	NA	NA	IV thiamine 200 mg, every 12 h, for 5 days	Mortality	Improvement in SOFA score (between day 1 and day 6)
Nandhini et al. (13)	Single-center randomized controlled trial	25/25	54/54	60/45	IV thiamine 2 mg/kg, every 8 h, for 3 days	Lactate (daily, for 3 days)	Dose and duration of vasopressor support SOFA score (daily, for 3 days) Need for RRT Hospital mortality

### 3.4. Summary of results

#### 3.4.1. Primary outcome: mortality

**Figure 2:**
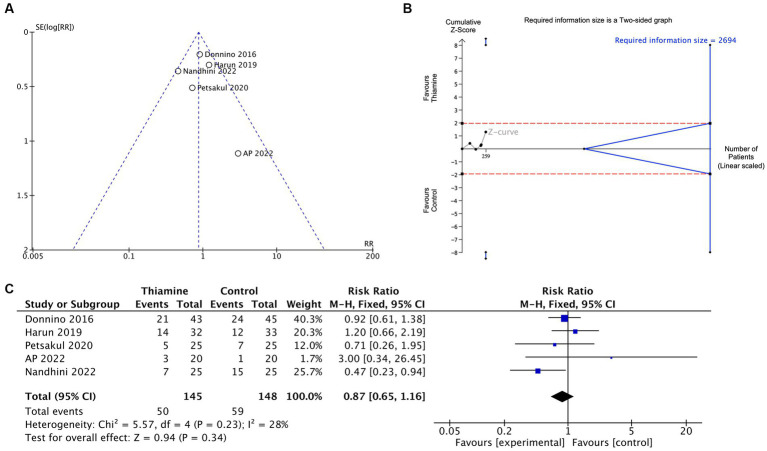
Effect of thiamine in septic shock on mortality. **(A)** Funnel plot showing the precision of the studies (Standard Error, SE) against the treatment effect as risk ratio (RR). **(B)** Trial sequential analysis showing the cumulative Z-score among included studies, according to the number of patients included. The horizontal lines represent the significant threshold with alpha set to 5% (two-sided test). The converged lines represent trial sequential boundaries (significance boundaries) adjusted in such a way that the total Type I and II errors remain at the level set in the sample size calculation. **(C)** Forest plot showing the mean difference in mortality.

The five studies that were included assessed mortality in 293 patients. The funnel plot for the primary outcome did not suggest publication bias (Figure 2A). Based on our pre-set criteria, the RIS was not reached (Figure 2B).

The incidence of mortality ranged from 5 to 60% (median 36%), and from 15 to 49% (median 28%) in the control and thiamine groups, respectively.

The intervention did not change mortality significantly RR 0.87 (95%CI 0.65; 1.16, *I*^2^ = 21%) *p* = 0.34 (Figure 2C).

#### 3.4.2. Secondary outcomes

**Figure 3:**
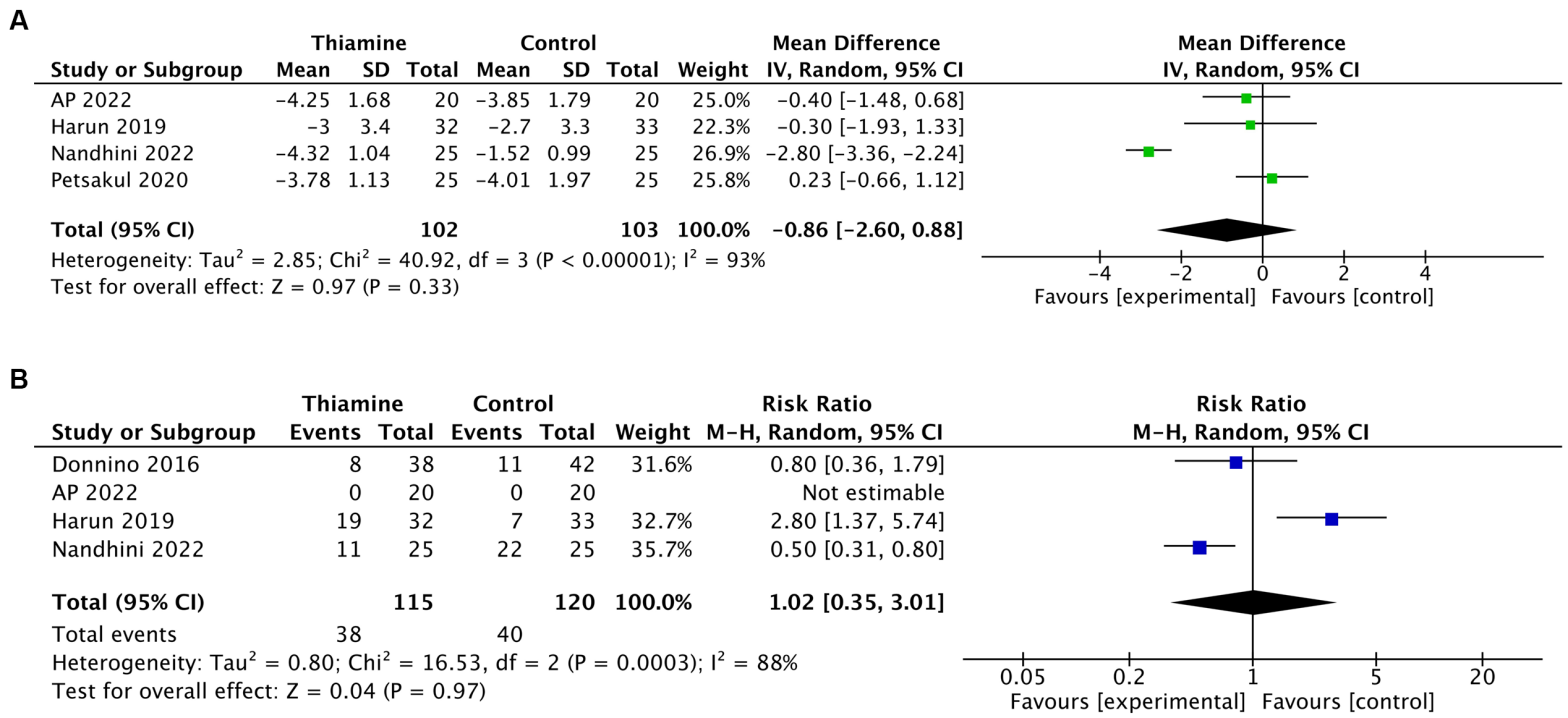
Effect of thiamine in septic shock on secondary outcomes. **(A,B)** Funnel plot showing the precision of the studies (Standard Error, SE) against the treatment effect as mean difference (MD) for SOFA reduction withn 4 days **(A)** and as risk ratio (RR) for need for RRT **(B)**.

SOFA reduction within 4 days and need for RRT were both available in 4 trials. Neither of these two outcomes significantly differed between the two groups [Mean Difference −0.86 (95% CI −2.60; 0.88, *p* = 0.33 and RR 1.02 95% CI 0.35, 3.01, *p* = 0.97 respectively)] (Figures 3A,B).

## 4. Discussion

In our meta-analyses, use of thiamine alone did not improve mortality in septic shock patients compared to standard care.

Thiamine is critical for mitochondrial function, acting as a cofactor for the Pyruvate Dehydrogenase Complex (PDH) and the Alpha-keto-glutarate dehydrogenase, two enzymes involved in the tricarboxylic acid cycle (14). Also, as recently reported, thiamine has a significant role in renal gluconeogenesis (15), a process associated with better ICU outcomes.

However, very few studies assessed the effect of a thiamine alone supplementation in septic patients. Therefore, our work analyzed only five RCTs. Most of the existing trials focused on the association of ascorbic acid and thiamine. This was mainly consequent to the publication of a retrospective before/after study which reported improvements in many outcomes including mortality when using an association between thiamine and ascorbic acid in septic shock patients (4). In this publication, the addition of ascorbic acid was justified by its role as a cofactor for the norepinephrine synthesis pathway, its antioxidant properties as well as a potential synergic effect between these components. However, RCTs conducted since then have not confirmed these results (5, 16, 17). Moreover, several publications have raised concerns about possible harmful effects associated with high dose vitamin C (13, 18).

To this date, only five RCTs investigating the effect of thiamine alone in septic shock patients have been published. Among them, only one showed a significant benefit on mortality.

The study of Harun et al. (10), which compared thiamine (600 mg/24 h) to a placebo, reported a non-significatively higher ICU mortality in the thiamine group as compared to placebo (44% vs. 36%). Likewise, the study of Ap et al. (12) reported a null mortality in both groups. However, both studies were limited by their sample size, recruiting 65 and 6 patients, respectively.

In their study involving 50 ICU patients in septic shock, Petsakul et al. (11) reported a non-significatively lower 28-day mortality in the thiamine group (20% vs. 28%, *p* = 0.741). Despite the authors failing to show a significant difference in mortality, they reported a higher reduction of the vasopressors depending index in patients treated by thiamine.

Nandhini et al. (13) included 50 septic shock patients and is the only RCT reporting a significant benefit of thiamine treatment on mortality. The authors reported decreased ICU mortality in the group treated with thiamine (2 mg/kg, 8 hourly, for 3 days) compared to the placebo (28% vs. 60%, *p* = 0.021).

Finally, Donnino included the largest number of patients (45 and 43 patients in the control and thiamine groups respectively) (9). When comparing an intravenous infusion of thiamine (200 mg/12 h) to a placebo, they found hospital mortality at 53 and 49% in the control and thiamine groups, respectively. Interestingly, Donnino et al. (9) reported that 35% of the patients were thiamine deficient at baseline. Therefore, considering the predefined subgroup of patients with thiamine deficiency, the authors reported a decreased mortality in those treated with thiamine (13% vs. 46%, *p* = 0.047).

These results suggest that thiamine supplementation could mainly be beneficial in thiamine deficient patients.

The prevalence of thiamine deficiency in critically ill patients has never been described (19, 20) and is associated with increased morbidity and mortality (21). Also, an experimental model of sepsis found that thiamine deficiency induced oxidative stress and an inflammatory response (22).

However, to our knowledge, no study has specifically assessed the effect of thiamine supplementation in septic shock patients with initial thiamine deficiency.

Our study has several limitations mostly due to methodological weaknesses of the original studies. Firstly, only five RCTs were included in our meta-analyses. Estimating between study heterogeneity can be difficult when the number of included studies is low, thus leading to biased effect estimates. However, we performed a Mantel–Haenszel method which has been validated for meta-analyses of a small number of available studies (23). Secondly, we did not search for unpublished data and therefore cannot exclude a publication bias, which tends to overestimate the beneficial effect of an intervention. Finally, no definitive conclusion can be drawn from these studies, when considering the small number of included patients in the analyzed RCTs, as shown by the TSA, and the effect size of the intervention. According to the TSA, to detect a relative 14% difference on mortality, a sample size of 2,239 patients is needed.

## 5. Conclusion

Our meta-analysis shows that an adjunctive treatment of thiamine in septic shock patients did not improve mortality. However, the small number and the intrinsic weaknesses of included RCTs means a definitive conclusion cannot be drawn. Therefore, further studies are necessary to assess a potential benefit of a thiamine supplementation in septic shock patients, especially in those presenting with a thiamine deficiency.

## Data availability statement

The raw data supporting the conclusions of this article will be made available by the authors, without undue reservation.

## Ethics statement

Ethical approval was not required for the study involving humans in accordance with the local legislation and institutional requirements. Written informed consent to participate in this study was not required from the participants or the participants’ legal guardians/next of kin in accordance with the national legislation and the institutional requirements.

## Author contributions

DL and FS: conceptualization and validation. DL, FS, and BA: methodology and formal analysis. DL, FS, TV, AF, TG, and SC: data curation. DL, FS, and TV: writing—original draft preparation. DL, FS, SC, and SS: writing—review and editing. DL: supervision. All authors contributed to the article and approved the submitted version.

## Funding

DL is supported by two young researcher grants from the Geneva University Hospitals (PRD 5-2020-I and PRD 4-2021-II). Open access funding by University of Geneva.

## Conflict of interest

The authors declare that the research was conducted in the absence of any commercial or financial relationships that could be construed as a potential conflict of interest.

## Publisher’s note

All claims expressed in this article are solely those of the authors and do not necessarily represent those of their affiliated organizations, or those of the publisher, the editors and the reviewers. Any product that may be evaluated in this article, or claim that may be made by its manufacturer, is not guaranteed or endorsed by the publisher.
