# Supplementary material for: Thiamine as a metabolic resuscitator in septic shock: a meta-analysis of randomized controlled trials with trial sequential analysis
Source: Front Med (Lausanne). 2023 Sep 13;10:1223862. doi: 10.3389/fmed.2023.1223862 (PMC10533915; doi:10.3389/fmed.2023.1223862)

Supplementary file 1: risk of bias assessment for including studies

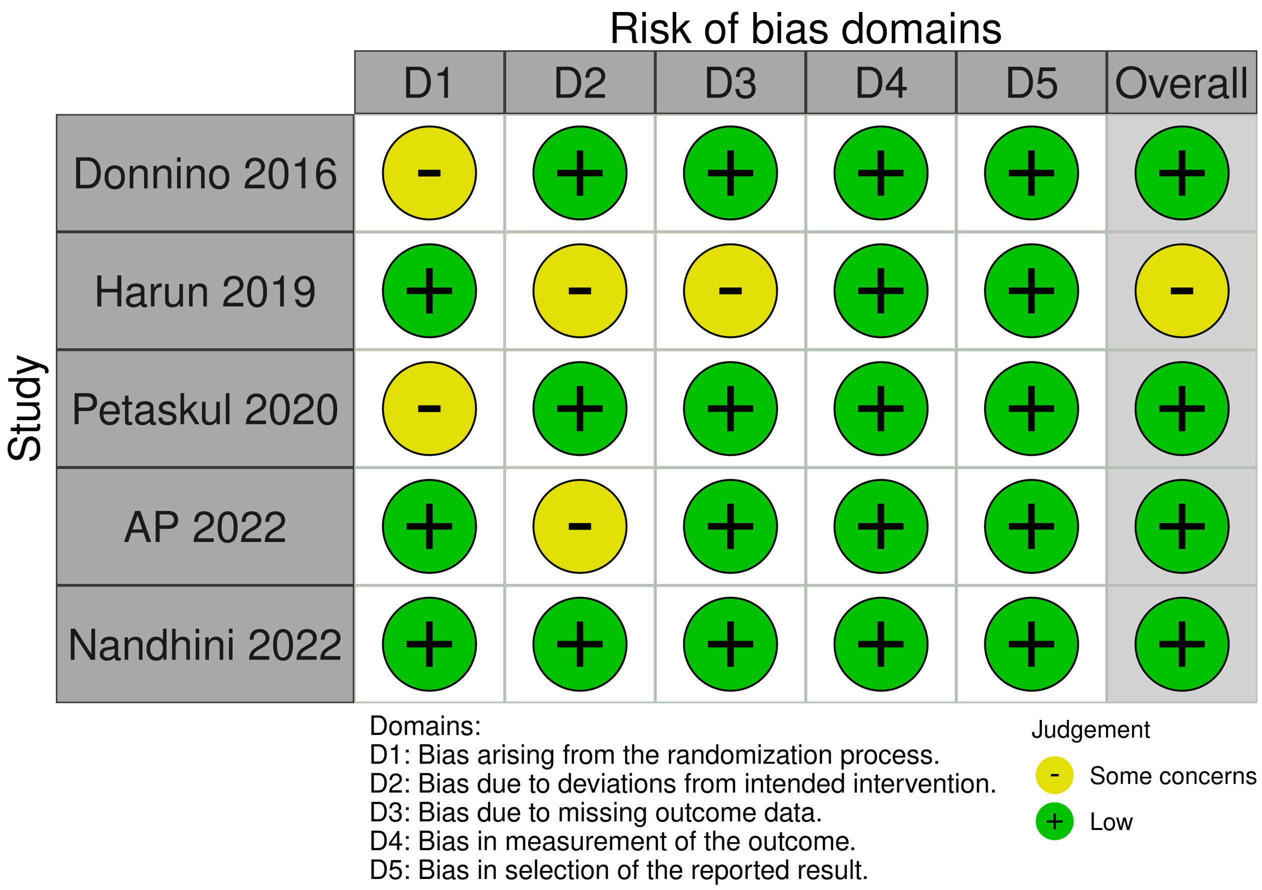

Supplement: Supplementary file 1 [file Image_1.pdf]
